# Isoflurane Postconditioning Upregulates Phosphorylated Connexin 43 in the Middle Cerebral Artery Occlusion Model and Is Probably Associated with the TGF-*β*1/Smad2/3 Signaling Pathway

**DOI:** 10.1155/2020/3451215

**Published:** 2020-03-17

**Authors:** Jiangwen Yin, Xuejiao Liu, Ruixue Wang, Mingyue Ge, Liping Xie, Jingwen Zhai, Zhigang Dai, Yan Li, Sheng Wang

**Affiliations:** ^1^Department of Anesthesiology, First Affiliated Hospital, School of Medicine, Shihezi University, Shihezi 832002, China; ^2^Department of Anesthesiology, The First Affiliated Hospital of Wenzhou Medical University, Wenzhou 325000, China; ^3^Department of Anesthesiology, First Affiliated Hospital of USTC, Division of Life Sciences and Medicine, University of Science and Technology of China, Hefei, Anhui 230001, China

## Abstract

**Aim:**

Connexin 43 (Cx43) has been identified to be important for cerebral ischemia/reperfusion (I/R) injury as well as protection from it. This study was aimed at investigating the relationship between phosphorylated Cx43 (p-Cx43), transforming growth factor-*β*1 (TGF-*β*1 (TGF-

**Methods:**

The middle cerebral artery occlusion (MCAO) model was induced in 96 male Sprague-Dawley rats, weighing 250-300 g. The rats were randomized into 12 groups, namely, sham, middle cerebral artery occlusion (MCAO)/I/R, I/R+1.5% ISPOC, I/R+LY2157299 (blocker of TGF-*β*1 (TGF-*β*1 (TGF-*β*1 (TGF-*β*1 (TGF-

**Results:**

Neurological deficit scores, brain infarct volume, and damaged neurons in the I/R group significantly increased compared to those in the sham group (*P* < 0.05). However, in the ISPOC group, damage of the brain was significantly ameliorated (*P* < 0.05). However, in the ISPOC group, damage of the brain was significantly ameliorated (*P* < 0.05). However, in the ISPOC group, damage of the brain was significantly ameliorated (*β*1 (TGF-*P* < 0.05). However, in the ISPOC group, damage of the brain was significantly ameliorated (*β*1 (TGF-*P* < 0.05). However, in the ISPOC group, damage of the brain was significantly ameliorated (*β*1 (TGF-*β*1 (TGF-*P* < 0.05). However, in the ISPOC group, damage of the brain was significantly ameliorated (

**Conclusion:**

Isoflurane postconditioning (ISPOC) may alleviate cerebral I/R injury through upregulating the expression of p-Cx43, and the TGF-*β*1/Smad2/3 signaling pathway may be involved in the process.*β*1 (TGF-

## 1. Introduction

Cerebrovascular disease is a group of diseases that are harmful to human health, and it has now become the main cause of human disability and death [1, 2]. Tissue injury caused by cerebral ischemia/reperfusion (I/R) is the main cause of stroke. Reperfusion of injured tissue after ischemia leads to neurologic damage, such as cell necrosis and apoptosis [3]. Although tissue plasminogen activator (tPA) is considered the gold standard treatment for ischemic stroke, one of its side effects is the enhancement of inflammatory response in brain capillaries and subsequently neuronal cell damage after stroke [4]. It has received extensive attention in clinical practice. Thus, finding a new treatment strategy is necessary.

Isoflurane (ISO) is a volatile anesthetic widely used in clinical practice, and it has been demonstrated to have neuroprotective effects on cerebral I/R injury [5–8]. Studies have indicated that ISO plays its role in neuroprotection by inhibiting cerebral I/R injury, which causes the decrease in neuronal death [9]. However, the specific mechanisms underlying the protective effects of ISO on cerebral I/R injury remain unclear. Our previous study showed that the appropriate concentration of ISPOC was 1.5% [10].

Connexins are proteins that are widely expressed in the body, and they are especially important for brain functions. Cx43 is one of the most common connexins in the brain, and it is crucially important for cerebral I/R injury [11]. Gap junctions (GJs) consist of connexin protein subunits with some head-to-head patterns, which provide the important channel for intercellular communication. In the brain, the GJ channels communicate various signals such as glucose, lactate, and Ca^2+^ between neurons to maintain the stability of the intracellular environment and other life processes [12]. Studies have shown that GJs induced cell apoptosis and mediate the spread of cell necrosis [13]. There is evidence that the phosphorylation of Cx43 also affects the function of GJ [14]. Protein kinase C can phosphorylate the Cx43 protein to inhibit the function of GJ [15, 16].

TGF-*β*1 is a well-known neuroprotective and neurotrophic factor in the central nervous system (CNS) and is involved in cerebral I/R injury [17]. Studies have shown that ISO plays its protective effect on cerebral I/R injury by increasing the expression level of TGF-*β*1 [10]. Furthermore, TGF-*β*1 can inhibit GJ function by altering the phosphorylation form of Cx43 [18, 19]. Numerous studies have reported that signaling pathways are involved in the change of neural function. In consideration of the role of Cx43 and TGF-*β*1 in the brain and the effects of isoflurane postconditioning (ISPOC), we hypothesized that ISPOC might exert its neuroprotective effect via upregulating the expression of p-Cx43, and the TGF-*β*1/Smad2/3 signaling pathway may be involved in this process. To verify this hypothesis, we used the middle cerebral artery occlusion (MCAO) model for the research.

## 2. Materials and Methods

### 2.1. Experimental Animals

Healthy adult male Sprague-Dawley rats (250-300 g) were provided by the Experimental Animal Center of Shihezi University. All rats were raised at the laboratory condition in a 12 h dark-light cycle with free access to food and water; the temperature is maintained at 20°C-25°C and the humidity at 55%-65%. Before this experiment, animals were all adaptively fed in the breeding environment mentioned above for at least one week. All animal procedures were approved by the Animal Care and Use Committee of the First Affiliated Hospital of the Medical College, Shihezi University, and were in accordance with the Guidance Suggestions for the Care and Use of Laboratory Animals, formulated by the Ministry of Science and Technology of China, and the *Guide for the Care and Use of Laboratory Animals* by the National Institutes of Health (NIH Publication No. 85-23, Revised in 2006).

### 2.2. Preparation for the Middle Cerebral Artery Occlusion (MCAO) Model

All the rats were anesthetized with a mixture of ketamine (60 mg/mL) and xylazine (10 mg/mL) by intraperitoneal injection (0.15 mL/100 g) and fixed on a thermostatic (37°C) operating table. The MCAO model was established with reference to the modified Longa method by making a middle incision on the neck and then separating the right common carotid artery (CCA), external carotid artery (ECA), and internal carotid artery (ICA). We ligated the ECA and CCA at the end near the heart using a 0.25 mm diameter nylon line (4-0, Ethicon, Japan) and inserted a small mouth cut in the CCA near the bifurcation. The insert depth was 18.0 ± 2.0 mm from CCA when a slight sense of resistance can be felt. Then, we trussed CCA and the nylon line inside. In the sham operation group, the line was inserted into CCA at the depth of 10 mm from bifurcation of CCA. Other procedures were similar to that of the experimental group. The 1% lidocaine was used by local injection around the incision for postoperative analgesia. After 90 min embolism, we pulled out the nylon line carefully to obtain the MCAO model. The rats in the inhibitor or activator group were injected with the TGF-*β*1 inhibitor LY2157299, p-Cx43 inhibitor Ro318220, p-Cx43 activator 18*β*-GA, or DMSO at the lateral ventricle of the brain stereo locator 30 min before the MCAO (coordinate localization: AP: B-0.8 mm, ML = ±1.5 mm lateral, and *H* = 4.0 mm depth). Both inhibitors and agonists were dissolved with 0.5% DMSO at 5 *μ*g/kg for a total injection of 20 *μ*L for 10 min; each side was injected for 5 minutes. To exclude the effect of solvent DMSO on isoflurane, rats in group 20 *μ*L of 0.5% DMSO solution were injected through the lateral ventricle of the brain stereo locator for 30 min before MCAO. MCAO and isoflurane postconditioning were routinely administered after injection.

### 2.3. Performance of Isoflurane Postconditioning

Experimental rats to be treated with 1.5% isoflurane postconditioning were placed in the inhalation anesthesia device (a self-made-patented product, patent number: ZL201620527058.6) after 90 min embolism (at the beginning of reperfusion). Then, we opened the compressed air tank and adjusted the flow to 1 L/min. The isoflurane evaporator is connected to the outlet of the compressed air and the inlet of the inhalation anesthesia box. Subsequently, we adjusted the evaporator scale to ensure that the isoflurane concentration is 1 minimum alveolar concentration (MAC) (1.5%). An anesthesia gas monitor (Dragger, Germany) was used to control and monitor the concentration of inhaled isoflurane. The posttreatment time for each group was 60 min. The rats were removed and returned to the cage for recovery at the end of postconditioning.

### 2.4. Neurologic Scoring

After 24 h of reperfusion, two observers who have no knowledge of the grouping were hired to assess the neurological deficits according to the 5-point scale by Longa et al. [20]. The average score of the two scorers was used for statistics. The scoring method is as follows: zero, no signs of neurologic damage in rats; one point, rats cannot fully extend the forepaw; two points, the rats circle to the opposite side; three points, the rats fall down to the opposite side; and four points, the rats cannot walk spontaneously, being unconscious. The premature dead rats were excluded from the experiment.

### 2.5. 2,3,5-Triphenyl Tetrazolium Chloride (TTC) Staining and Measurement of Infarct Volume

After assessment of neurological deficits (24 h of reperfusion), we anesthetized the rats for brain sampling by decapitation. We irrigated the residual blood with saline and took them for quick freezing (approximately 15 min) in a -20°C refrigerator. The brains were rapidly cut into five 2 mm thick coronal sections, and the sections were subsequently stained with 2% TTC (Sigma-Aldrich, St. Louis, MO, USA) for 20 min in a 37°C incubator, which was away from light. The 4% paraformaldehyde (PFA) was used to fix the brain tissue for at least 24 h. A digital camera was used to photograph the posterior surface of each slice. The image processing software (Image Pro-Plus 6.0) was used to measure the area of cerebral infarction (the red areas represent normal brain tissue and the pale areas represent the infarct area). The infarct volume was then calculated as a percentage of the infarct area relative to the contralateral hemisphere area in each slice.

### 2.6. Hematoxylin-Eosin (HE) Staining

HE staining was used to count the number of the damaged neurons. Before the staining, the rat hippocampal slices were fixed in 4% PFA for at least 72 h. First, the rat hippocampal slices were paraffin-embedded and then cut into 4 mm thick slices by using a semiautomatic skiving machine (KEDEE, KD-2850, China). After the above steps, the rats were finally stained with hematoxylin-eosin. With HE staining, the damaged neurons were characterized by contraction of the nucleus, cellular edema, vacuolization, and darkened nuclei. The light microscope (Nikon, Japan) was used to obtain the image [21].

### 2.7. Nissl Staining

According to the Nissl staining kit for experiment, the brain tissue samples were dewaxed with xylene for 45 min, dehydrated with the concentration of gradient ethanol, and then placed in the Nissl staining solution. Subsequently, the slices were rinsed with distilled water. Ethanol of 95% was used to spot color, and xylene was used to provide a transparent visual field. A neutral gum seal was used to seal them [22].

### 2.8. Terminal Deoxynucleotidyl Transferase dUTP Nick-End Labeling (TUNEL) Assay

The TUNEL method was used to evaluate apoptosis-associated DNA fragmentation by using the In Situ Cell Death Detection Kit (Roche Molecular Biochemical, Mannheim, Germany). The coronal sections were stained in accordance with the manufacturer's protocol. After staining, the TUNEL-positive neurons were stained dark brown, which can be easily observed, and the brain tissue slices were collected under an optical microscope. Each slice was randomly selected at five different visual fields under a 200x field of view. The image analysis system (Image Pro-Plus 6.0) was used to carry out positive cell analysis. A reticle was used to count cells in the same size area.

### 2.9. Quantitative Real-Time Polymerase Chain Reaction (qRT-PCR)

The nucleotide sequence was retrieved in GenBank, and Premier 5.0 software was used for primer design of the target genes. The extraction of total ribonucleic acid (RNA) in the hippocampus was carried out using the Bizol kit (Bioer, BSC51M1, Hangzhou, China). The total RNA content was measured by means of a micronucleic acid protein analyzer (Thermo Fisher, USA). Then, the RevertAid First Strand cDNA Synthesis Kit (Bioer, BSB09M1, Hangzhou, China) was used to reverse the total RNA into cDNA. RT-PCR was also conducted using the SYBR Green I Real-time PCR Kit (Bioer, BSB03L1, Hangzhou, China). The primers we used are presented in Table 1. ABI 7500 (Applied Biosystems, 7500 Fast, USA) was used for the quantitative PCR, and the reaction conditions were as follows: 94°C for 2 min, followed by 40 cycles at 94°C for 10 s and 60°C for 30 s, at which point data were acquired. The Ct value we obtained is the number of reactions, and a small Ct value indicated less reaction times. This finding means that the original expression of the target gene increased. This detection index was analyzed by the 2^−ΔΔCt^ method. In addition, each target gene was subjected to three repeat holes, and each sample was repeated thrice.

### 2.10. Immunofluorescence (IF) Staining

Rat hippocampal tissue sections were dewaxed with concentration gradient xylene for 45 min (15 min for each concentration). After dewaxing, the sections were washed with phosphate-buffered saline (PBS) for 5 min thrice. Citrate buffer (pH = 6.0) was used for antigen retrieval by a microwave for 12 min. Sections were then washed with PBS for another three times and incubated for 2 h with a mixture containing 5% bovine serum albumin (BSA), PBS, and 0.3% Triton X-100 at room temperature (25°C-30°C). These tissue sections were then incubated at 4°C for at least 12 h with an anti-Cx43 (phospho-S368) primary antibody (1 : 50, Abcam, rabbit, ab194928). The sections were subsequently incubated with a fluorescein isothiocyanate secondary antibody (1 : 100, ZSGBBIO, goat, ZF-0311) for 45 min at room temperature being protected from light. Propidium iodide (PI) solution (0.5 *μ*g/mL) was used for cell nucleus staining (30 s-1 min). After the above steps, we observed the distribution and expression level of the target protein in cells by laser scanning confocal microscopy (Zeiss LSM 510, Germany) at a corresponding wavelength. For each section, we randomly selected five different visions to collect pictures under a 200x view field. Fluorescence intensity was then measured by an image examiner image analysis system. Mean fluorescence intensity (MFI) was used to indicate the expression level of proteins.

### 2.11. Western Blot Analysis

We mixed the hippocampus tissue with radioimmunoprecipitation assay (RIPA) lysis buffer (1 mL RIPA+10 *μ*L phenylmethylsulfonyl fluoride, Wuhan Boster Bioengineering, China), and then, the mixture was disrupted by a Sonifier disruptor and lysed in an ice box for 30 min. The concentration of the protein samples was measured with an ultra-microspectrophotometer (NanoDrop 2000, Thermo, USA). The total proteins were separated by 10% sodium dodecyl sulfate polyacrylamide gel electrophoresis and transferred into a polyvinylidene fluoride membrane (Solarbio, China) in a wet turn membrane apparatus and then incubated with 5% evaporated milk or BSA as a blocking reagent (1 g of nonfat milk, 20 mL of 1× Tris-buffered saline with Tween 20 (TBST)) for 2 h. The primary antibodies were as follows: anti-TGF-*β*1 (1 : 1000, Abcam, rabbit, ab92486), anti-Cx43 (1 : 1000, Abcam, rabbit, ab11370), anti-Cx43 (phosphor-S368) (1 : 500, Abcam, rabbit, ab194928), anti-*β*-actin (1 : 1500, ZSGB-BIO, mouse, sc-47778), anti-Smad2/3 (1 : 1000, Abcam, ab63672), and p-Smad2/3 (1 : 1000, Abcam, ab63399). The primary antibodies were all incubated for 12 h at 4°C. Then, we washed the membranes thrice with TBST. A secondary antibody (1 : 10,000, ZSGB-BIO, goat, ZB2301, China) was incubated for 45 min at room temperature (25°C-30°C). An enhanced chemiluminescence detection kit (Thermo Fisher Scientific, 34094, China) was used to show the blots following the information in the manual. To analyze the results, we used a mean value of three experiments.

### 2.12. Statistical Analysis

Mean ± standard deviation (SD) was used to represent all measurement data. Multivariate-repeated measurement analysis of variance (ANOVA) was used in comparison among groups. Post hoc Bonferroni's test was used when conducting multiple comparisons. One-way ANOVA and Dunnett's *t*-test were used for Western blot data analysis. All the data were statistically analyzed by SPSS 20.0 software. *P* < 0.05 was considered statistically significant.

## 3. Results

### 3.1. Effects of 1.5% ISPOC on Neurological Deficit Scores in Rats with Cerebral I/R Injury

**Figure 1:**
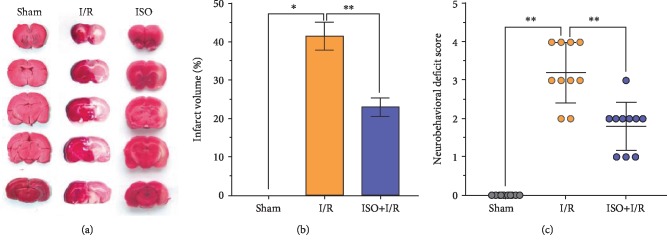
Effects of 1.5% ISPOC on neurological deficit scores and infarct volume on cerebral I/R injury. (a) Brain sections (2 mm thick) were stained with 2% TTC. The red-stained area indicates normal areas, and the pale area signifies ischemic areas of the brain tissue. TTC: 2,3,5-triphenyltetrazolium chloride. (b) Proportion of the brain infarct volume in the ipsilateral hemisphere. The results are expressed as means ± standard error of the mean (SEM) (*n* = 8). ^∗^*P* < 0.05, ^∗∗^*P* < 0.01. (c) Neurological deficit scores were assessed after middle cerebral artery occlusion (MACO) for 90 min and reperfusion for 24 h. The results are presented in a scatter plot format (*n* = 10). ^∗^*P* < 0.05, ^∗∗^*P* < 0.01.

The neurological deficit scores of all the experimental rats were normal (0 point) before cerebral I/R injury. After 24 h of reperfusion, neurological deficit scores of the I/R group significantly increased compared with those of the sham group (*P* < 0.01 vs. sham group), but this situation was significantly ameliorated through 1.5% ISPOC (*P* < 0.01 vs. I/R group, Figure 1(c)).

### 3.2. Effects of 1.5% ISPOC on the Cerebral Infarct Volume in Rats with Cerebral I/R Injury (TTC Staining)

In addition to neurological deficit scores, the protective effects of 1.5% ISPOC were evaluated through measuring the cerebral infarct volume. No infarcted areas were observed in the sham group. Conversely, obvious infarcted areas were observed in the MCAO model group (I/R group). However, the 1.5% ISPOC group exhibited a significantly smaller infarct volume compared with the I/R group (*P* < 0.01, Figures 1(a) and 1(b)).

### 3.3. Effects of 1.5% ISPOC, TGF-*β*1 Inhibitor LY2157299, p-Cx43 Inhibitor Ro318220, and p-Cx43 Activator 18*β*-GA on the Survival of Hippocampal CA1 Neurons after Cerebral I/R Injury in Rats (HE Staining)

**Figure 2:**
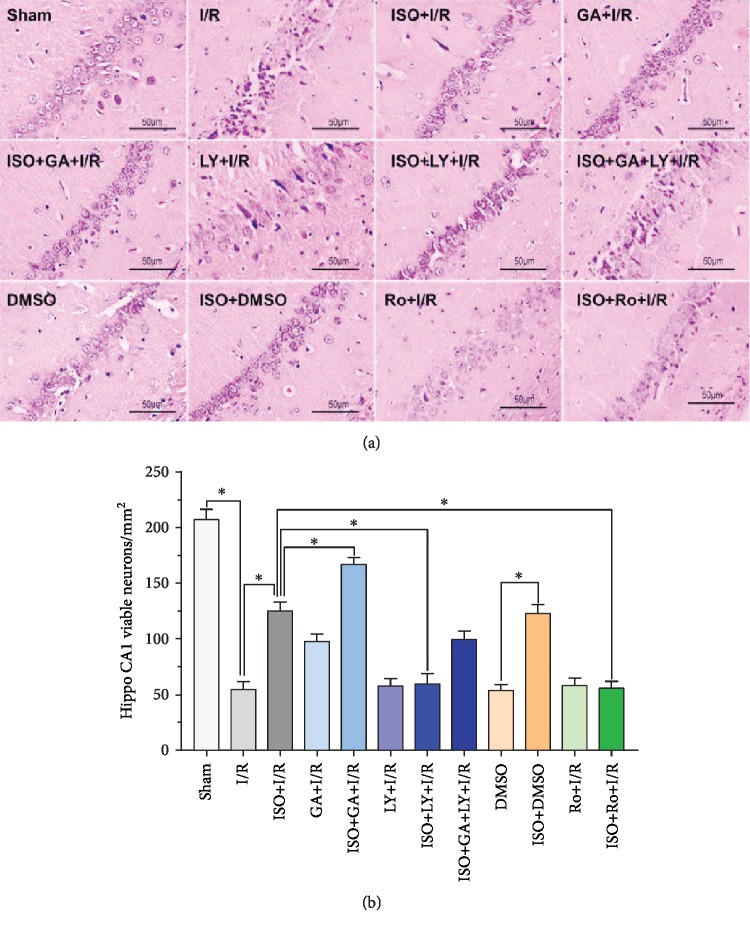
Effects of 1.5% ISPOC, TGF-*β*1 inhibitor LY2157299, p-Cx43 inhibitor Ro318220, and p-Cx43 activator 18*β*-GA on the survival of hippocampal CA1 neurons after cerebral I/R injury in rats (HE staining). (a) Brain sections were stained with HE. Contraction of the nucleus and cellular edema showed the impaired neurons in the hippocampal CA1 area. Original magnification: 200x. Scale bars: 50 *μ*m. (b) Proportion of injured neurons in the CA1 area. The results are expressed as the means ± SEM (*n* = 8). ^∗^*P* < 0.05, ^∗∗^*P* < 0.01.

With HE staining, we observed that in the sham-operated group, almost no neurons were damaged in the hippocampal CA1 region and the proportion of injured neurons was 0.5 ± 0.1%. In the MCAO group, the number of neurons decreased significantly and the proportion of injured neurons was 68.7 ± 2.4%. However, the necrotic cells in the 1.5% ISPOC group were significantly fewer than those in the I/R group, and the proportion of injured neurons was 37.9 ± 1.2%. After applying the TGF-*β*1 inhibitor LY2157299, the number of necrotic cells significantly increased and the ratio of injured neurons was 65.7 ± 1.8%. When pretreated with the p-Cx43 inhibitor Ro318220 before reperfusion, neuronal damage induced by ischemia was obviously aggravated and the proportion of damaged neurons was 72.3 ± 3.6%. On the contrary, after using the p-Cx43 activator 18*β*-GA, the number of damaged cells significantly reduced and the proportion of injured neurons was 17.8 ± 1.1% (Figures 2(a) and 2(b)).

### 3.4. Effects of 1.5% ISPOC, TGF-*β*1 Inhibitor LY2157299, p-Cx43 Inhibitor Ro318220, and p-Cx43 Activator 18*β*-GA on Neuronal Morphology in the Hippocampal CA1 Region after Cerebral Ischemia/Reperfusion in Rats (Nissl Staining)

**Figure 3:**
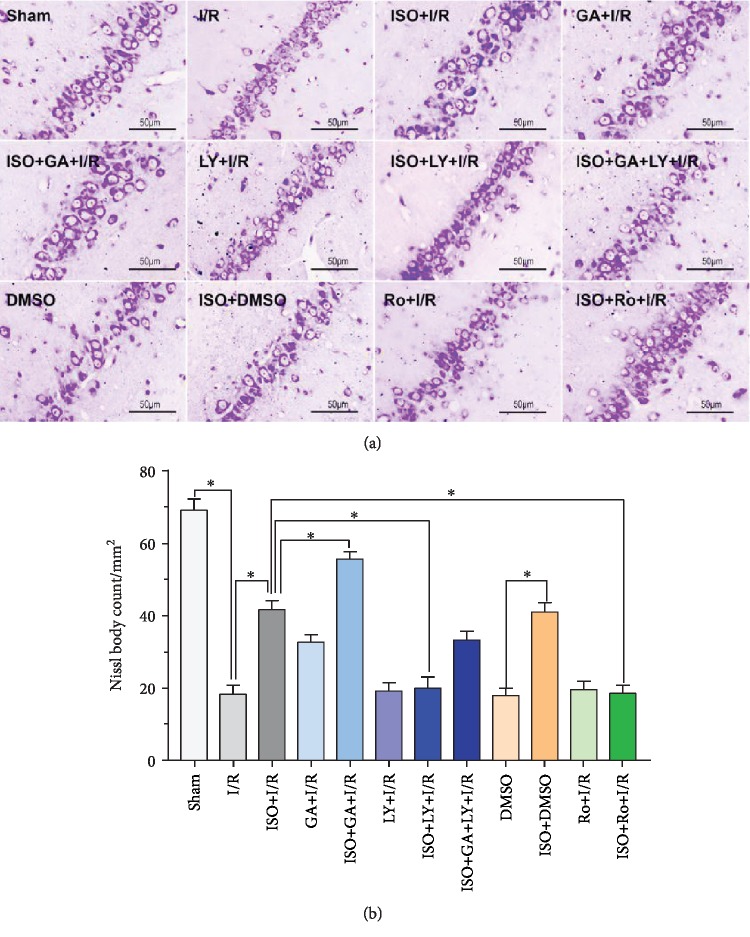
Effects of 1.5% ISPOC, TGF-*β*1 inhibitor LY2157299, p-Cx43 inhibitor Ro318220, and p-Cx43 activator 18*β*-GA on neuronal damage in the hippocampal CA1 region after cerebral ischemia/reperfusion (Nissl staining). (a) Brain sections were stained with the Nissl staining solution. The number of Nissl bodies in the hippocampal CA1 area neurons showed the extent of damage. Original magnification: 200x. Scale bars: 50 *μ*m. (b) Number of Nissl bodies in the CA1 area in each group. The results are expressed as means ± SEM (*n* = 6). ^∗^*P* < 0.05, ^∗∗^*P* < 0.01.

Nissl staining showed that in the MCAO group (24 h after reperfusion), positive cells of Nissl staining in the hippocampus CA1 region significantly decreased compared with those in the sham group (*P* < 0.05), whereas 1.5% ISPOC can markedly increase the number of positive cells in the CA1 area of the hippocampus (*P* < 0.05 vs. the MCAO group). However, Nissl bodies were significantly reduced after the application of the TGF-*β*1 inhibitor LY2157299 (*P* < 0.01 vs. 1.5% ISPOC). When pretreated with the inhibitor of p-Cx43 (Ro318220), positive cells of Nissl staining remarkably decreased compared with those treated with 1.5% ISPOC (*P* < 0.01). The number of Nissl staining-positive cells increased significantly after the application of 18*β*-GA (*P* < 0.05 vs. MCAO group). No significant difference was observed between the DMSO group and MCAO group (*P* > 0.05, Figures 3(a) and 3(b)).

### 3.5. Effects of 1.5% ISPOC, LY2157299, Ro318220, and 18*β*-GA on Apoptosis of Hippocampal CA1 Region Cells in Rats with Cerebral I/R Injury (TUNEL Assay)

**Figure 4:**
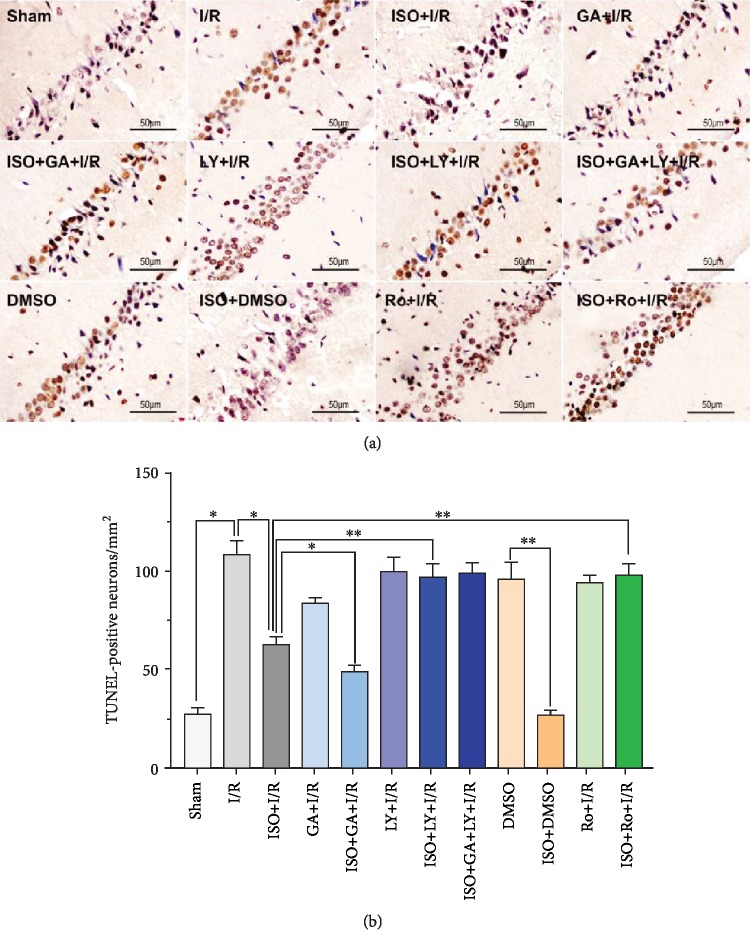
Effects of 1.5% ISPOC, LY2157299, Ro318220, and GA on TUNEL-positive cells in the hippocampal CA1 area after cerebral I/R injury. (a) TUNEL staining exhibited a strong color reaction (dark brown), and accurate distribution of apoptotic cells can be observed under the light microscope. The density of TUNEL-positive cells was determined by two researchers who were blinded to the grouping settings. Original magnification: 200x. Scale bars: 50 *μ*m. (b) Data are expressed as the means ± SEM (*n* = 8). ^∗^*P* < 0.05, ^∗∗^*P* < 0.01.

The apoptotic cells in the hippocampus significantly increased in the MCAO group compared with the sham group (*P* < 0.05). However, the 1.5% ISPOC significantly reduced the number of positive apoptotic cells in the hippocampus CA1 region (*P* < 0.05), whereas when pretreated with Ro318220 (the inhibitor of p-Cx43) before reperfusion, apoptotic cells induced by ischemia increased clearly (*P* < 0.01). Meanwhile, when LY2157299 (the TGF-*β*1 blocker) was injected, the neuroprotective effect of ISPOC was abolished (*P* < 0.01). When treated with the p-Cx43 activator GA, the number of positive apoptotic cells in the hippocampal CA1 region decreased markedly (*P* < 0.05, Figures 4(a) and 4(b)).

### 3.6. Expression Levels of TGF-*β*1 and p-Smad2/3 Protein after 1.5% ISPOC in Rats with Cerebral I/R Injury

**Figure 5:**
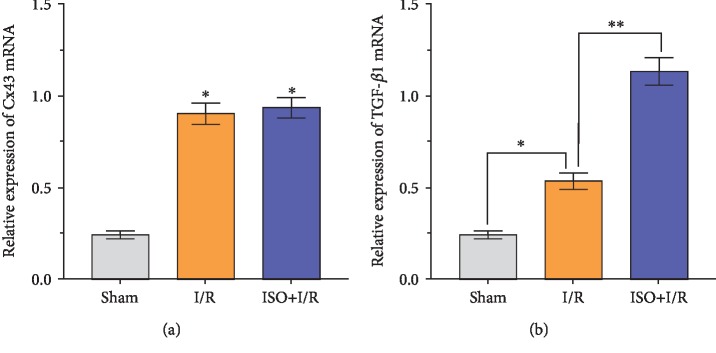
mRNA synthesis level of TGF-*β*1 and Cx43 was quantitated by the 2^−ΔΔCt^ method, and *β*-actin was used as a reference. The total ribonucleic acid (RNA) was extracted from the hippocampus tissue. (a) Expression of Cx43 mRNA in the ISO group has no significant difference from that in the I/R group. (b) Expression of TGF-*β*1 mRNA in the ISO group was higher than that in the I/R group. ^∗^*P* < 0.05, ^∗∗^*P* < 0.01.

**Figure 6:**
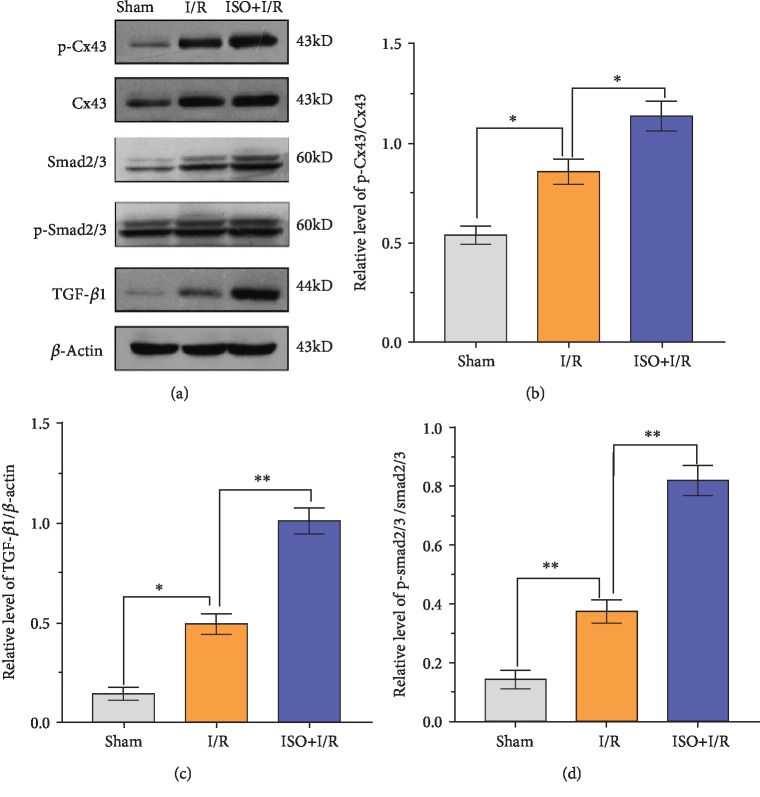
Relationship between the expression levels of phosphorylated Cx43 and TGF-*β*1 protein in rats with cerebral ischemia/reperfusion injury. Western blot tests for p-Cx43, Cx43, Smad2/3, p-Smad2/3, and TGF-*β*1 protein expression in each group. The grey level represents the expression level of each protein. *β*-Actin was used as an internal control. Dunnett's *t*-test was used for data analysis. The data are expressed as the means ± SEM (*n* = 6). ^∗^*P* < 0.05, ^∗∗^*P* < 0.01.

**Figure 7:**
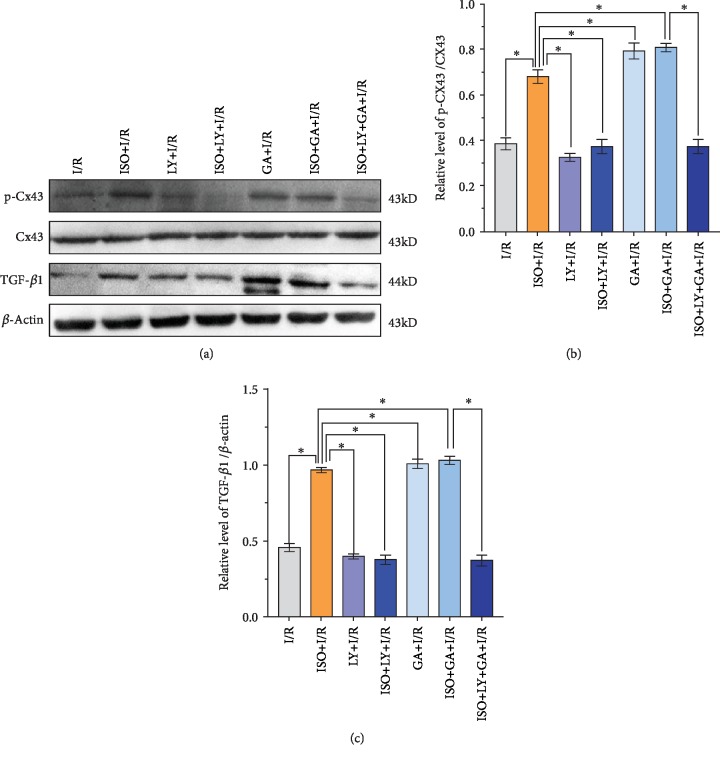
Expression of p-Cx43, Cx43, and TGF-*β*1 in the hippocampal tissues when the LY2157299 and 18*β*-GA on 1.5% ISPOC were administered to rats with cerebral ischemia/reperfusion injury. (a) Western blot analysis presented the expression of p-Cx43, Cx43, and TGF-*β*1 in the hippocampal tissues. *β*-Actin was used as an internal control. *n* = 10 rats per group. (b) The expression pattern of the phosphorylated Cx43 was synchronized with that of TGF-*β*1 during the entire process, whereas the total Cx43 protein expression levels did not change in all groups. Dunnett's *t*-test was used for data analysis. The data are all expressed as the means ± SEM (*n* = 6). ^∗^*P* < 0.05, ^∗∗^*P* < 0.01.

**Figure 8:**
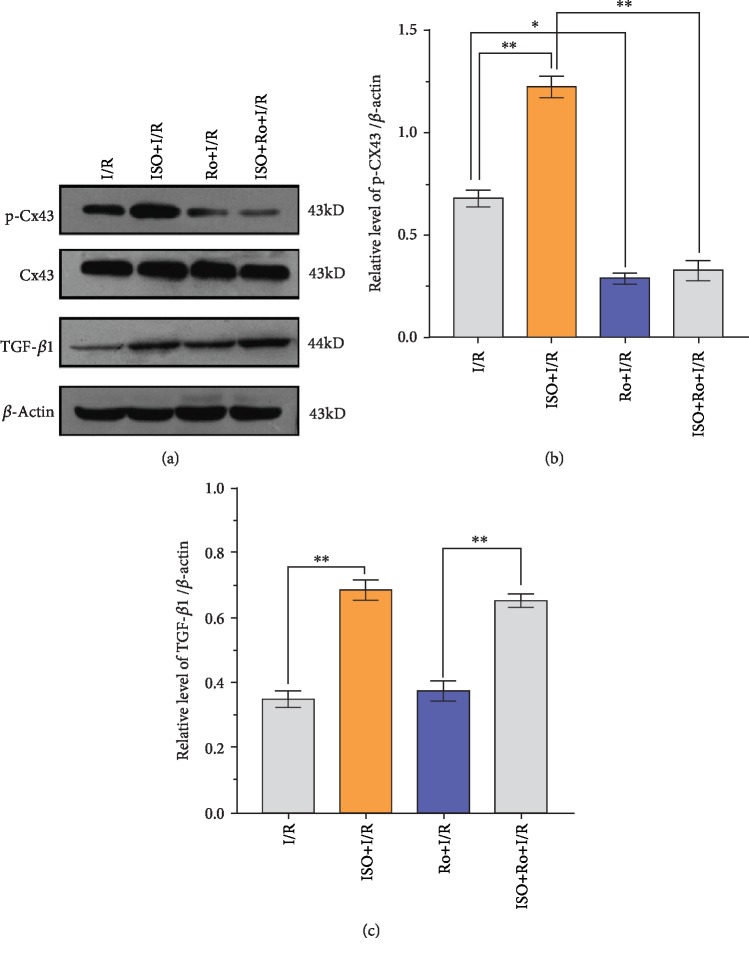
Western blot analysis presented the expression of p-Cx43, Cx43, and TGF-*β*1 in the hippocampal tissues when the inhibitor of p-Cx43 (Ro318220) is administered. *β*-Actin was used as an internal control. *n* = 10 rats per group. The expression pattern of the phosphorylated Cx43 was synchronized with that of TGF-*β*1 during the entire process, whereas the total Cx43 protein expression levels did not change in all groups. Dunnett's *t*-test was used for data analysis. The data are all expressed as the means ± SEM (*n* = 6). ^∗^*P* < 0.05, ^∗∗^*P* < 0.01.

Western blot analysis and qRT-PCR showed a light expression of TGF-*β*1 in the hippocampus tissue of the I/R group. Compared with the I/R group, the expression level of TGF-*β*1 protein increased significantly in the ISPOC group (*P* < 0.01, Figures 5(b) and 6). However, in the LY2157299+I/R and LY2157299+ISO+I/R groups, the expression of TGF-*β*1 decreased significantly (*P* < 0.05 vs. I/R group). Furthermore, the expression of total Smad2/3 protein was almost unchanged in all groups, but p-Smad2/3 showed a synchronous change with TGF-*β*1 (Figure 7). On the contrary, the expression of TGF-*β*1 and p-Smad2/3 did not change significantly in the Ro318220+ISO+I/R group (*P* > 0.05 vs. I/R group, Figure 8).

### 3.7. Changes of the Expression Levels of Cx43 and Phosphorylated Cx43 after 1.5% ISPOC in Rats with Cerebral I/R Injury

**Figure 9:**
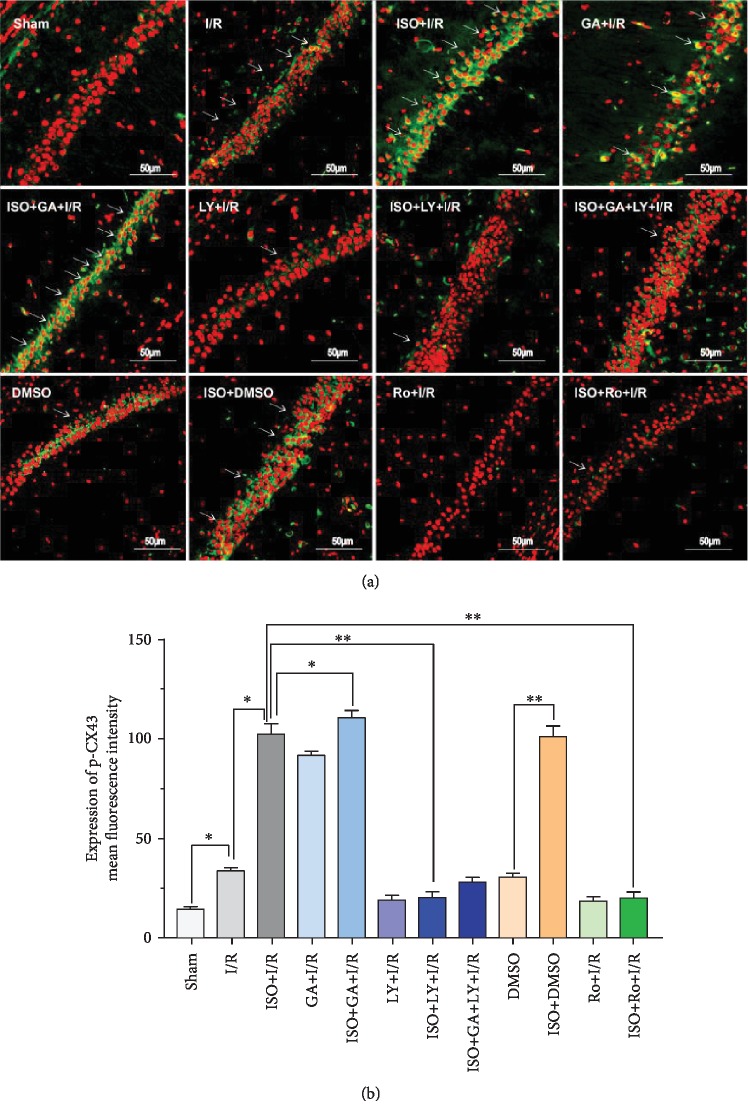
Relationship between p-Cx43 and TGF-*β*1/Smad2/3 signaling pathway after 1.5% ISPOC in rats with cerebral I/R injury. (a) IF showed fluorescence intensity of p-Cx43 after using the p-Cx43 inhibitor Ro318220 and the blocker of TGF-*β*1 (original magnification: 200x; ⟶ indicates positions of p-Cx43). (b) Mean fluorescence intensity was used to represent the relative expression level of p-Cx43 after immunofluorescence staining. ^∗^*P* < 0.05, ^∗∗^*P* < 0.01.

Western blot analysis, immunofluorescence, and qRT-PCR showed changes of Cx43 and phosphorylated Cx43 between groups. Compared with the sham group, the expression level of the phosphorylated Cx43 protein in the MCAO (I/R) group significantly increased (*P* < 0.05) but was lower than that in the ISPOC group (*P* < 0.05, Figure 6). However, when treated with the inhibitor of p-Cx43 (Ro318220), the expression of p-Cx43 in the ISPOC group significantly decreased (*P* < 0.01 vs. I/R group, Figures 8(a), 9(a), and 9(b)) and the protective effect of ISPOC was counteracted by Ro318220 (*P* < 0.05, Figures 2, 3, 4, 8, and 9). Nevertheless, no significant changes were observed in the expression of total Cx43 protein in all groups (*P* > 0.05, Figures 5(b), 6, 8, and 7).

### 3.8. Relationship between p-Cx43 and TGF-*β*1/Smad2/3 Signaling Pathway after 1.5% ISPOC in Rats with Cerebral I/R Injury

In this study, we explored the expression of p-Cx43 and TGF-*β*1 by using qRT-PCR, immunofluorescence, and Western blot. Immunofluorescence showed that in the hippocampal CA1 region, the majority of p-Cx43 were expressed in pyramidal neurons and the mean fluorescence intensity (MFI) of these proteins in the ISPOC group was much stronger than that in the I/R group (*P* < 0.05, Figures 9(a) and 9(b)). Results of Western blot indicated that expression of TGF-*β*1 in rats with I/R injury was markedly higher than that in the sham group (*P* < 0.05) but lower than that in the ISPOC group (*P* < 0.05, Figures 6(a) and 6(c)). The expression pattern of p-Smad2/3 was similar to that of TGF-*β*1 (Figure 6(d)), whereas the total Smad2/3 did not change in the three groups in the whole process (*P* > 0.05). Expression of TGF-*β*1 mRNA in hippocampal tissue of rats also confirmed TGF-*β*1 expression in the perilesional area of the I/R group in comparison with the corresponding area in the ISPOC group (*P* < 0.01) (Figure 5(b)). ISPOC apparently upregulated the expression level of p-Cx43 after cerebral I/R injury in comparison with the I/R group (*P* < 0.05, Figures 6(a) and 6(b)). Furthermore, the expression levels of TGF-*β*1 in the hippocampus of rats were increased by ISPOC compared to those in the I/R group (*P* < 0.05). However, with the treatment of the TGF-*β*1 inhibitor, LY2157299, the upregulated effects of ISPOC on TGF-*β*1 and p-Cx43 were all abolished (*P* < 0.05). Conversely, when treated with Ro318220, the p-Cx43 inhibitor, the expression level of TGF-*β*1 did not change significantly, which showed the potential interplay between the TGF-*β*1/Smad2/3 signaling pathway and p-Cx43 in the process of ISPOC against cerebral I/R injury. The protective effect of ISPOC against neuronal apoptosis in the CA1 area was blunted by LY2157299 (*P* < 0.05, Figures 2, 3, 4, 8, 7, and 9).

## 4. Discussion

Isoflurane (ISO) has been widely used in clinical anesthesia, and it is an ideal inhalation anesthetic. As an anesthetic, isoflurane has been studied by many researchers on the neuroprotective agents and mechanisms of ischemic brain injury, and isoflurane induces a postconditioning effect in the CNS [23, 24]. Nevertheless, the reaction of brain cells to injury is a dynamic process. Our previous studies demonstrated that isoflurane postconditioning displays a protective effect on cerebral I/R injury [10]. The application results of low, medium, and high concentration of ISO (1, 2, and 3 MAC) on rats that received 90 min ischemia and underwent isoflurane postconditioning for 60 min after reperfusion showed that 1 MAC isoflurane postconditioning significantly decreased the infarct volume and improved the neurological behavior score. However, when the concentration of isoflurane was increased to 3 MAC, this protective effect was not obvious. Therefore, the appropriate concentration (1 MAC) was used to further study the neuroprotective effect of isoflurane in this study. In the present study, the results of the HE and TUNEL methods all showed that 1 MAC (1.5%) ISPOC could significantly reduce the number of apoptotic cells in the hippocampal CA1 area of the injured side after cerebral I/R injury. Through TTC staining, we observed that 1 MAC ISPOC can significantly ameliorate infarct volumes in the brain and reduce the neurobehavioral scores in rats after cerebral I/R injury at the same time. Neuroprotective effects of 1 MAC ISPOC were also shown in Nissl staining, and therefore, we were convinced that 1.5% ISPOC can assuredly ameliorate cerebral I/R injury.

Among all the connexin protein subunits, Cx43 is the most abundant and common connexin protein in the brain and it is the primary component protein in gap junctions, which allow the passage of ions and small molecules [25]. The phosphorylation of Cx43 can regulate the permeability of gap junctions (GJ); thus, Cx43 phosphorylation is very important for its biological effect [26]. p-Cx43 has been shown to play an important role in cerebral I/R injury [27]. Another research showed enhanced expression of p-Cx43, but not total Cx43 in cerebral I/R injury [28]. In addition, other neuroprotective factors, such as nerve growth factor, blunt the negative effects caused by ischemic events by promoting Cx43 phosphorylation [29]. At the same time, different trends of phosphorylation and Cx43 dephosphorylation were also observed in the same drug [30]. In view of p-Cx43 being closely related to the function of gap junctions, we hypothesize that p-Cx43 may be involved in the neuroprotective effect of ISPOC. In the present study, our results showed that ISPOC upregulated the expression of p-Cx43. However, when the p-Cx43 inhibitor (Ro318220) was administered, the protective effects of ISPOC against neuronal apoptosis in the CA1 region were blunted. These findings indicate involvement of p-Cx43 in ISPOC against cerebral I/R injury in rats.

The expression of TGF-*β*1 in CNS contributes to the proliferation and differentiation of neurons in the brain [31–33]. Studies have also shown that TGF-*β*1 upregulates Cx43 expression in human trophoblast cells mediated by the Smad2/3 and ERK1/2 signaling pathways [34]. Furthermore, TGF-*β*1 was proven to be a regulator of Cx43, and it affects the intercellular communication mediated by GJ by modulating the Cx43 expression [35]. To our knowledge, only a minority of studies explored the expression of Cx43 mediated by the TGF-*β* signaling pathway to date in cerebral I/R injury. Our previous study demonstrated that ISPOC can upregulate the expression of TGF-*β*1 and the downstream p-Smad2/3 [10], which indicated that the TGF-*β*1/Smad2/3 signaling pathway may be involved in the neuroprotective effects of ISPOC and play a potential role in the mediation of p-Cx43 during ISPOC. Studies have shown that the activation of Smad2 and Smad3 is involved in the Cx43 expression induced by TGF-*β*1 [36]. In this study, we indeed found enhanced expression of TGF-*β*1 and p-Smad2/3 in ISPOC against cerebral I/R injury. In our study, we investigated the effects of LY2157299, a blocker of TGF-*β*1, on the expression of p-Cx43 after ISPOC. Studies have shown that the exposure to volatile anesthetics (isoflurane, sevoflurane) caused membrane externalization of PS detected by positive annexin V staining and increased the release of TGF-*β*1 into the cell culture media. Exogenous TGF-*β*1 induced protection, and a neutralizing TGF-*β*1 antibody prevented the cytoprotection by volatile anesthetics against hydrogen peroxide-induced cell necrosis. Volatile anesthetics induce a cytoprotective signaling cascade via membrane externalization of PS initiating TGF-*β*1-mediated cytoprotection [37], which indicates the direct link between isoflurane treatment and TGF-*β*1. Our results verified that ISPOC can upregulate the expression of TGF-*β*1, p-Smad2/3, and p-Cx43, whereas the total Smad2/3 and total Cx43 had no change. The application of LY2157299 decreased the expression of p-Smad2/3 and counteracted the neuroprotective effects of ISPOC. These results proved that the TGF-*β*1/Smad2/3 signaling pathway plays an effective role in the neuroprotective effects of ISPOC. At the same time, LY2157299 reduces the expression level of p-Cx43. By contrast, expression levels of TGF-*β*1 and p-Smad2/3 did not decrease significantly with preinjection of the p-Cx43 inhibitor (Ro318220), which showed the potential interplay between the TGF-*β*1/Smad2/3 signaling pathway and p-Cx43 in the process of ISPOC against cerebral I/R injury.

## Figures and Tables

**Table 1:** Primer sequences used in qRT-PCR.

Genes	Forward	Reverse
Cx43	5′-AGCCTGAACTCTCATTTTTCCTT-3′	5′-CCATGTCTGGGCACCTCT-3′
TGF-*β*1	5′-TGCTTCAGCTCCACAGAGAA-3′	5′-TGGTTGTAGAGGGCAAGGAC-3′
*β*-Actin	5′-AGGGAAATCGTGCGTGACAT-3′	5′-GAACCGCTCATTGCCGATAG-3′

1Cx43: connexin 43; TGF-*β*1: transforming growth factor beta-1.

## Data Availability

The raw/processed data required to reproduce these findings cannot be shared at this time as the data also forms part of an ongoing study.
